# Oscillatory Regulation of Hes1: Discrete Stochastic Delay Modelling and Simulation

**DOI:** 10.1371/journal.pcbi.0020117

**Published:** 2006-09-08

**Authors:** Manuel Barrio, Kevin Burrage, André Leier, Tianhai Tian

**Affiliations:** 1 Departamento de Informática, Universidad de Valladolid, Valladolid, Spain; 2 Advanced Computational Modelling Centre, University of Queensland, Brisbane, Australia; University of Auckland, New Zealand

## Abstract

Discrete stochastic simulations are a powerful tool for understanding the dynamics of chemical kinetics when there are small-to-moderate numbers of certain molecular species. In this paper we introduce delays into the stochastic simulation algorithm, thus mimicking delays associated with transcription and translation. We then show that this process may well explain more faithfully than continuous deterministic models the observed sustained oscillations in expression levels of *hes1* mRNA and Hes1 protein.

## Synopsis

Delay processes are ubiquitous in the biological sciences but are not always well-represented in mathematical models attempting to describe these biological processes. Additional issues arise when attempting to capture the uncertainty (intrinsic noise) associated with chemical kinetics in dealing with when and in what order reactions take place. Complicating the situation further are important instances when certain key molecules occur only in small numbers, so that it is not meaningful to talk about concentrations.

In this paper Barrio et al. show how to incorporate delay, intrinsic noise, and discreteness associated with chemical kinetic systems into a very simple algorithm called the delay stochastic simulation algorithm (DSSA). This algorithm very naturally generalises the stochastic simulation algorithm that does not treat delays. The authors then apply the DSSA to a specific set of experiments performed by Hirata et al. who showed, amongst other things, that serum treatment of cultured cells induces cyclic expression of both mRNA and protein of the Notch effector Hes1 with a two-hour period. The authors show how this approach can explain additional experiments performed by Hirata et al., and, because this approach is very general, suggest that it can provide deep insights into the relationship between delayed processes, intrinsic noise, and small numbers of molecules in many biological systems.

## Introduction

The mathematical modelling and simulation of genetic regulatory networks can provide insights into the complicated biological and chemical processes associated with genetic regulation. However, it is important that the models are kept simple but nevertheless capture the key processes. In addition, by incorporating experimental data into such models (where available) their accuracy can be improved.

An important aspect associated with genetic regulation is that mRNA and protein expression levels can be quite low, and so continuous models, as described by ordinary differential equations, may be inappropriate. Furthermore, processes such as transcription and translation do not occur instantaneously and may have considerable delays associated with them. It is these two issues that we pursue in terms of understanding oscillatory expression levels of both mRNA and protein of the Notch effector Hes1.

There are many types of molecular clocks that regulate biological processes, but apart from circadian clocks [1] these clocks are still relatively poorly characterised. Oscillatory dynamics are also known from mRNAs for Notch-signalling molecules such as Hes1 (a bHLH factor) that oscillates with a two-hour cycle during somite segmentation [2]. In a recent set of experiments, Hirata et al. [3] measured the production of *hes1* mRNA (*M*) and Hes1 protein (*P*) in mouse. Serum treatments on cultured cells (that have already been shown to induce circadian oscillation [4]) result in oscillations in expression levels for *hes1* mRNA and Hes1 protein in a two-hour cycle with a phase lag of approximately 15 min between the oscillatory profiles of mRNA and protein. The oscillations in expression continue for 6–12 h and are not dependent on the stimulus but can be induced by exposure to cells expressing Delta. It was argued that the lag between protein and mRNA oscillation levels of 15 min reflects the time needed for protein degradation.

Specifically, the data presented in the paper by Hirata et al. (Figure 1 in [3]) indicates sustained oscillation of *hes1* mRNA over six periods while it suggests oscillation of Hes1 protein that dies away after 6–8 h. Furthermore, the peaks in the expression levels of Hes1 protein seem to be about twice that of the mRNA peaks. Unfortunately, it is not clear what the units are in terms of expression levels and whether the scales are the same from experiment to experiment—valuable information that might help to validate a mathematical model.

**Figure 1:**
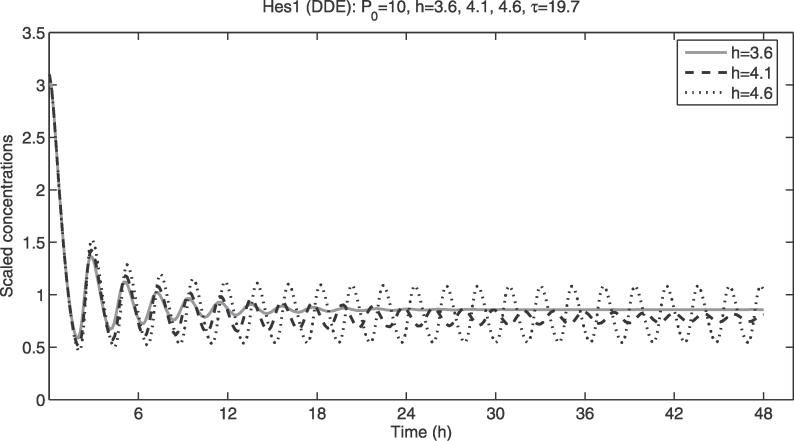
DDE Solution: Exploring the Effect of *h* on Protein

Hirata et al. examined the underlying mechanisms for the observed oscillations and showed that in the presence of the proteasome inhibitor MG132, *hes1* mRNA is initially induced but after 3 h it is suppressed because of constant repression of transcription by persistently high protein levels (negative autoregulation). Treatment with cycloheximide leads to sustained increase of *hes1* mRNA and blocks its oscillation. A similar effect occurs with overexpression of dnHes1, a dominant-negative form of Hes1 that is known to suppress Hes1 protein activity [5]. These results reveal that both Hes1 protein synthesis and degradation are needed for oscillations in the expression levels of *hes1* mRNA. Other experiments showed that the same mechanisms hold for *hes1* mRNA expression levels in the presomitic mesoderm in mouse. Interestingly, it is known that in mouse presomitic mesoderm the expression levels of other signalling molecules such as *lnfg* also oscillate [6]. However, *lnfg* is not expressed in the cultured cells of Hirata et al., indicating that serum-induced Hes1 oscillation does not depend on *lnfg*. Nevertheless, this does suggest that Hes1 and *lnfg* oscillations are controlled by a similar mechanism. Hirata et al. estimate the half-lives of *hes1* mRNA and Hes1 protein to be 24.1 ± 1.7 min, 22.3 ± 3.1 min, respectively. Experiments with various protease inhibitors suggest that Hes1 protein is specifically degraded by the ubiquitin–proteasome pathway. They also lower the temperature in their experiments from 37 °C to 30 °C, which lowers both the synthesis and degradation rates, and this alters the period of the oscillation (unpublished data).

To explain the observed behaviour, Hirata et al. modify a mathematical model developed by Elowitz and Leibler [7] for a synthetic gene network constructed in Escherichia coli cells by introducing one gene from *λ*-phage. By postulating a Hes1 interacting factor as a third molecular species, they obtain a system of three ODEs that give rise to sustained oscillatory behaviour. However, there is no direct experimental evidence for such an interacting factor. Rather, the introduction of a third variable is due to the fact that certain systems of two ODEs cannot generate sustained oscillations.

This observation together with the experimental results of Hirata et al. led to a number of papers in which simple coupled delay differential equations (DDEs) representing *M* and P were developed to explain the sustained oscillations without recourse to the addition of a third variable (Monk [8], Jensen et al. [9], Lewis [10], and Bernard et al. [11]).

In fact, one of the first people to consider feedback differential equation models for the regulation of enzyme synthesis was Goodwin [12]. U. an der Heiden [13] modified these ideas by including transport delays into Goodwin's model. The oscillatory behaviour of the ensuing DDEs as a function of the size of delays was investigated by an der Heiden.

The ideas underpinning these works are that the processes of transcription, translation, and export are not instantaneous. Monk notes that there is an average delay of 10–20 min between the action of a transcription factor on the promoter region of a gene and the appearance of the corresponding mRNA in the cytosol. Similarly, there is a delay of typically 1–3 min for the translation of a protein from mRNA. Note that the model proposed by Lewis is for zebrafish but it does offer insights into the Hes1 mechanisms via the general nature of the model.

These papers were able to explain some of the observed experimental results quite well, but there are still some aspects that these delay continuous models do not address. These aspects relate to the fact that production numbers of mRNA and protein can be quite low and that intrinsic noise effects due to the uncertainty in knowing when a reaction and what reaction takes place in any given time interval can be very important. Thus the aim of this paper is to incorporate delay effects into the discrete stochastic simulation algorithm (SSA) of Gillespie [14] and to see whether the dynamical behaviour of this delay stochastic simulation algorithm (DSSA) can give greater insights into the nature of *hes1* mRNA and Hes1 protein in mouse and, by extension, to other genetic regulatory networks.

## Methods

Let *M*(*t*) and *P*(*t*) represent the concentrations of *hes1* mRNA and Hes1 protein, respectively. Absorbing all the delays (including transcription and translation) into one delay, *τ,* the model presented in Monk [8] and Jensen et al. [9] is represented by the following DDE:

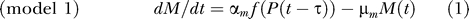


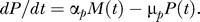



Here *μ_m_* and *μ_p_* are the degradation rates for *M* and *P, α_m_* is the maximal mRNA transcription rate in the absence of protein repression, and *α_p_* is the translation rate, while *f*(*P*(*t*)) is a monotonically decreasing Hill function representing the repression of mRNA production by the binding of Hes1 dimers to the promoter region. It takes the form

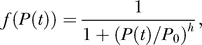
where *h* is the Hill coefficient representing the cooperative character of the binding process and *P*_0_ is such that *f*(*P*_0_) = 1/2.

Bernard et al. [11] give a modification to model 1 by arguing that the Hirata et al. observations could be better explained by the existence of an additional agent in the Hes1 repression loop. A new variable *Q*(*t*) represents the concentration of a repression complex of hyper-phosphorylated Gro/TLE1 with Hes1 protein. This leads to the delay model

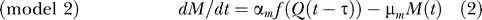


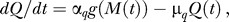
where *f* is as before, *α_q_* is the maximal phosphorylation rate, and

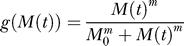
is a monotonically increasing Hill function representing the Gro/TLE1 protein activation.

The astute reader might ask what happens if the transcriptional and translational delays, *τ_m_* and *τ_p_,* respectively, are not lumped together. This would lead to

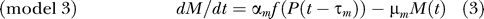


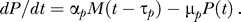



But if we let *N*(*t*) = *M*(*t* – *τ_m_*), which is a phase-lagged mRNA variable, then we can always write model 3 in the form of model 1 with *τ* = *τ_p_* + *τ_m_*. Thus, it is sufficient to consider model 1 (Lewis [10] noted this relationship when he used the delay model 3 to describe alternating bands of gene expression that distinguish anterior and posterior components of somites in zebrafish).

Monk and Jensen et al. investigate the dynamics of model 1, especially in terms of the onset of sustained oscillations, through simulations; while Bernard et al. give a mathematical investigation of the dynamics of models 1 and 2 by a scaling process and performing a linear stability analysis around the steady state values. Lewis shows that, given *h* = 2, model 3 will have sustained oscillations if

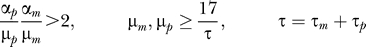
and that the period of the oscillations is approximately

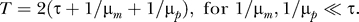



Bernard et al. have given a detailed mathematical bifurcation analysis of the dynamics of the models 1 and 2. In the case of model 1 let the time period of the oscillations be T and define

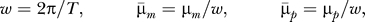
then sustained oscillations will occur if

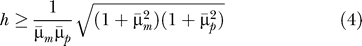


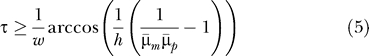
where they have used a scaling such that with

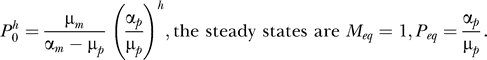



It is clear that h, *μ_m_,* and *μ_p_* have crucial roles in the onset of sustained oscillations, while *α_m_, α_p_,* and *α*_0_ play no significant role. Using the data from the Hirata et al. experiments, we can now see how well model 1 matches the observed results.

### Model Analysis

We now give a brief analysis of model 1 and see how well it can describe the results of Hirata et al. and what sort of predictions it can make.

#### The relationship between *h* and *τ*.

Using some of the values in Table 1, Bernard et al. have shown that sustained oscillations can be obtained with *h* ∈ (3,7), *τ* ∈ (15,30), and *T* ∈ (90,150). For the experimentally observed period *T* = 120 min, then *h* ≥ 4.1, *τ* ≥ 19.7. On the other hand, Hes1 is a dimer, but there are at least three separate binding sites for Hes1 dimers in the regulatory region of the *hes1* gene, so that an appropriate value of *h* is at least 2. However, whether *h* should be as large as 4.1, as this model predicts, is debatable. We show later in this paper that *h* need not be that large to get sustained oscillations when discrete models are used.

**Table 1:**
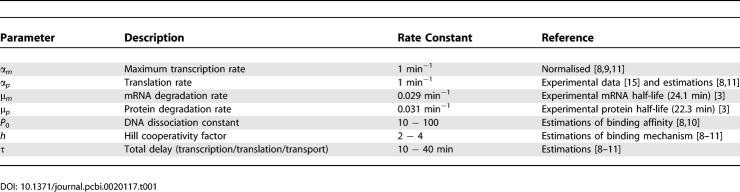
Model Parameters Used for DDE and DSSA

Jensen et al. show via simulations that for the case *h* = 2, oscillations are only sustained for *τ* > 80 and there are no oscillations for *τ* < 10. For *τ* ∈ (10,80), the period of the damped oscillations is approximately 170 min, which is much greater than the observed period of 120 min.

#### Parameter sensitivity.

Simulations and mathematical analysis show that there is no qualitative difference in terms of the onset of sustained oscillations and their period for a wide range of values for *α_m_, α_p_,* and *P*_0_. Monk has performed a linear stability analysis and has shown that for a certain range (that is quite wide) of these parameters the oscillatory period is approximately constant.

It is interesting to note that when Hirata et al. lowered the temperature from 37 °C to 30 °C there was a change in the period of oscillation, but as the data is not given we are unable to test these effects in terms of model 1 except to reaffirm that the model is not sensitive to the production terms.

More significantly, in the continuous model, the Hes1 protein concentration rarely falls below the repression threshold *P*_0_, which means that Hes1 transcription is always repressed. While this does not contradict experimental data, as there is no mention of this threshold, it does mean that there is not a strong link between the continuous model and the actual mechanism of transcription.

#### Overshoot.

Bernard et al. note that systems with just one nonlinear term often display large overshoot before solutions converge to an attractor. Indeed, that is one of the reasons why they introduce model 2. They attempt to estimate this overshoot, which is essentially due to the lack of repression mechanisms in the first few minutes. Defining the overshoot to be the ratio of the protein concentration at *t* = *τ* to its steady state value, then under the assumptions that

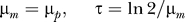
the expression for overshoot is

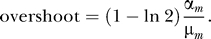



With *α_m_* = 1, *μ_m_* = 0.03, this implies an overshoot of approximately 11.

However, the overshoot only becomes an issue with simulations if the initial conditions are set close to zero. Monk avoids this large overshoot in his simulations by setting the initial conditions close to their steady state values. The real issue here, however, is of course how the oscillations are set off within a cell by serum treatment. The Hirata data does not show any overshoot and so there is a need to relate the initial conditions of any model to the experiment itself. Inevitably, this treatment will change one or more of the model parameters, perhaps continuously. Thus more work needs to be done to understand how serum treatment induces oscillations before we can address this issue more appropriately from a modelling perspective.

#### Peak-to-trough ratio.

Deterministic, continuous models do not match very well the peak-to-trough ratios observed by Hirata et al. Indeed, for model 1 this ratio is higher for mRNA than protein, and this appears to contradict the Hirata data.

#### Experimental validation.

We acknowledge that it is often hard to compare experimental and simulated results for the purposes of model validation. However, we note that Hirata et al. performed some experiments in terms of blocking protein degradation and translation, and while model 1 has not been tested in this regard we do attempt to mimic these experimental results in this present paper through our use of discrete models.

In summary, model 1 predicts possibly high Hill factors, overshoot (if the initial conditions are not chosen very carefully), and no obvious link between the values of *P*_0_ and the actual physical basis of transcription. The model is also very sensitive to the degradation parameters but not sensitive to the production parameters. Thus, it can explain some (but not all) of the Hirata data.

However, there are two fundamental issues that the model does not address and which could well explain some of the discrepancies mentioned above. These are that mRNA and proteins can be expressed in quite small numbers and that there is intrinsic noise in terms of the uncertainty of knowing when a certain reaction and what reaction takes place. These points lead us into a discussion on discrete stochastic models for chemical kinetics and the SSA. This in turn will lead to the main idea of this paper, namely the incorporation of delays into discrete, stochastic models and how this approach may address the issues raised here.

### Discrete, Stochastic Models

Key molecules that are produced at low levels and a chemical systems' intrinsic noise led to Gillespie (1977) [14] introducing the SSA, which describes the evolution of a discrete, stochastic chemical kinetic process in a well-stirred mixture:

Let there be *m* chemical reactions between *N* chemical species inside some fixed volume *V* held at constant temperature. The reactions can be uniquely characterised by the *m* stoichiometric vectors *v*_1_,…,*v*_m_, and propensity functions *a*_1_(*X*),*a*_2_(*X*),…,*a*_m_(*X*). Here *X*(*t*) is the vector of chemical species (*X*_1_(*t*),…,*X_N_*(*t*))*^T^,* where *X_i_*(*t*) is the number of molecules of species *i* at time *t*. The propensity functions represent unscaled probabilities of a particular reaction taking place. More formally, *a_j_*(*X*(*t*))*dt* represents the probability of reaction *j* occurring within the time interval (*t,t* + *dt*). For elementary kinetics the propensity functions take very simple forms. For instance, for the second order reaction *X*_1_ + *X*_2_ 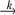 *X*_3_, it is *a*(*X*) *= kX*_1_(*t*)*X_2_*(*t*). Similar formulas apply for unimolecular and dimer reactions. The evolution of *X* through time can be considered to be a discrete nonlinear Markov process that is described by the SSA.

The underlying idea behind the SSA is that at each time point *t* a step size *θ* is determined from an exponential waiting time distribution such that at most one reaction can occur in the time interval (*t*,*t* + *θ*). If the most likely reaction, as determined from the relative sizes of the propensity functions, is reaction *j*, say, then the state vector is updated as

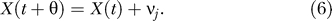



See Algorithm 1 for a pseudo-code description of the SSA.

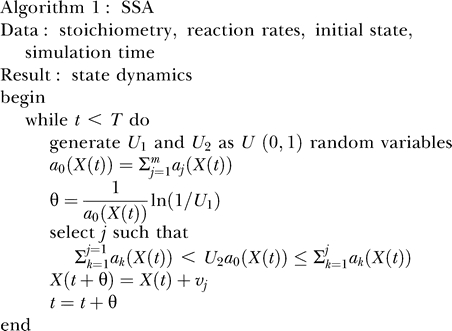



The SSA has been used successfully in many settings (e.g., Arkin et al. [16] for the study of *λ*-phage). But its limitation is that it can be very computationally expensive, as large numbers of simulations are needed to calculate moments of *X*(*t*) accurately and because the time step can become very small. For this reason a number of approaches have been recently developed to improve the performance of SSA. These include the Poisson leap method (Gillespie [17]) and the Binomial leap method (Tian and Burrage [18]) in which larger time steps are allowed so that all reactions can fire in that step with a frequency sampled from a Poisson or Binomial distribution, respectively.

Other approaches for improving the performance of SSA are based on the chemical master equation (CME) that describes the evolution of the probability density function *p*(*X*,*t*) such that

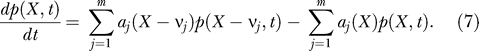



It is possible to cast this problem into the form *dp*/*dt* = *Ap* where *A* is the state space matrix, which can be enormous. To make this problem more computationally tractable, quasi–steady state assumptions can be used (where possible) to either reduce the size of the problem or to partition the problem into different regimes with different methods being applied (Haseltine and Rawlings [19], Goutsias [20], Burrage et al. [21]).

We note that an approximation to the mean behaviour *μ*(*t*) = *E*[*X*(*t*)] can be derived from Equation 7 to give

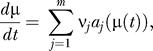
which is the standard chemical kinetics rate order equation for describing concentrations. This can be seen by multiplying both sides by *X*(*t*) and summing over all possible configurations of the state space (see [22]).

Given this overview of SSA, our intention is now to introduce delays into SSA and to investigate the dynamics of model 1 in this setting. Unlike the SSA, there is not necessarily a unique implementation of delay SSA (DSSA), and issues pertaining to this are discussed in more detail in Text S1.

Briefly, DSSA implementations can differ in the way they handle (1) the waiting time for delayed reactions, (2) the time steps in the presence of delayed reaction updates, and (3) delayed consuming reactions. The DSSA version we used to produce the results presented in the following section works as follows: initially we specify which nonconsuming reactions are delayed and the delay size (constant or variable) associated with each reaction. Delayed consuming reactions are not allowed. Simulations proceed by drawing reactions and their waiting times (for delayed and nondelayed reactions). If a nondelayed reaction is selected, then the state is updated in the standard way (SSA), but if it is a delayed reaction that is selected then it is not updated until the appropriate time point would be passed by another simulation step. In this case, the last drawn reaction is ignored and instead the state is updated according to the delayed reaction. Simulation continues at the corresponding time point. Algorithm 2 shows a pseudo-code description of the DSSA implementation.

In general, delays in time evolutions are difficult to handle because of the non-Markovian character they introduce into the dynamical process. In this context we note that our DSSA implementation ignores the elapsed time between the last triggered reaction and the update of the next scheduled delayed reaction. It is unclear whether this affects the distribution of waiting times until the next reaction happens. It also ignores the selected reaction that should be updated beyond the current update point by preferentially updating the delayed reaction. However, it is an open question whether we should select for the delayed reaction and ignore the other. For further discussions we refer the reader to Text S1.

Furthermore, we note that as soon as we introduce delays into SSA then the evolution of *X*(*t*) is no longer described by a Markovian process and the nature of the CME in this case needs further consideration. We have made additional material available in Text S2 in which we derive from first principles a CME for the DSSA. It generalises Equation 7 in a very natural manner. Having constructed the CME for the delay case, we can then multiply both sides by all possible configurations of the state space and this will lead to a DDE for the mean (see Text S2 for further details).

Model 1 can be presented in DSSA form with four reactions defined by

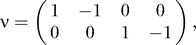


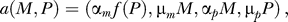
with the delay occurring in the first reaction.

We note that the time step we use for DSSA is self-selecting based on the assumption of exponential waiting times, as is the case for SSA. The stiffer the kinetics system becomes (due to large rate constants and/or large numbers of molecules), the smaller the time step. Thus, the algorithm intrinsically controls the stability of the evolution. However, in the case of the continuous DDE representation, an important issue is stepsize selection for any numerical method to avoid instabilities in the computed solutions.

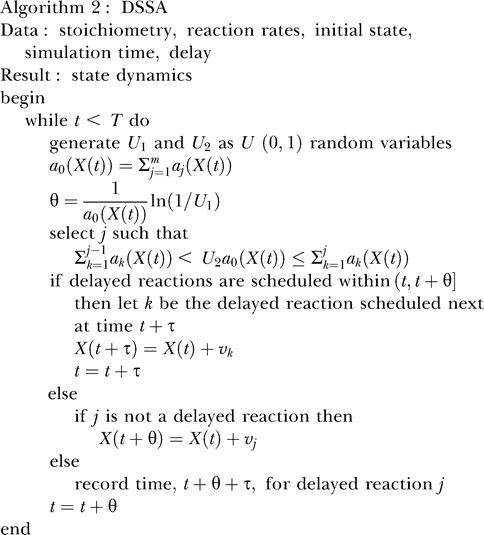



## Results

### Parameter Exploration and Model Comparison

In this section we present a selection of DDE solutions and DSSA trajectories displaying the dynamical properties of model 1. As for the DSSA, what we present are single simulations of just one particular strong solution based on a particular path generated by the random variables. Nevertheless, these individual solutions are very representative of the dynamics of the processes being modelled. In some cases, we perform a number of independent simulations to collect information about mean behaviour.

**Figure 2:**
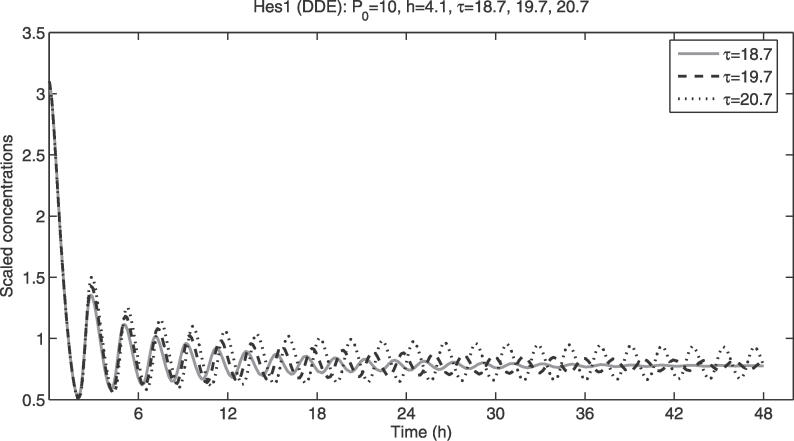
DDE Solution: Exploring the Effect of Delay on Protein

**Figure 3:**
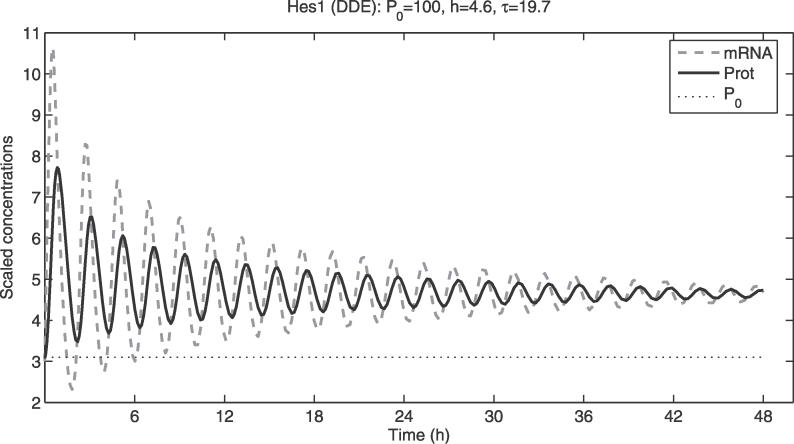
DDE Solution: Exploring the Effect of Large *P*_0_

All DDE plots were generated using the *dde23* function in MatLab. The initial conditions are set to (*M*(0),*P*(0)) = (3,100). In Figures 1–3 we have scaled the protein concentrations by *μ_p_* so that both mRNA and protein numbers fit conveniently on the same figure. Figures 1 and 2 show the dynamics of the continuous DDE model for protein concentration with *P*_0_ = 10, *h* = (4.6,4.1,3.6), *τ* = 19.7 and *P*_0_ = 10, *h* = 4.1, *τ* = (20.7,19.7,18.7), respectively.

In Figure 1 the delay is kept fixed and the Hill factor *h* varies around the bifurcation point 4.1. We clearly see that for *h* = 3.6 the oscillations damp quickly, while for *h* = 4.6 the oscillations are sustained. In Figure 2 the Hill factor is kept fixed and instead the delay varies around the bifurcation point 19.7. We observe a similar behaviour as in Figure 1, namely that for *τ =* 18.7 the oscillations damp quickly, while for *τ =* 20.7 the oscillations are sustained. Interestingly, however, the size of *P*_0_ can affect these dynamics. For values of *τ =* 18.7 up to about 10 there is no essential difference when the sustained oscillations arise. In Figure 3 both the concentrations of mRNA and protein are plotted. We see that when *P*_0_ is increased to 100 oscillations damp for values of *h* greater than the critical value of 4.1, namely for (*h*,*τ*) = (4.6,19.7). In this case the concentrations of *P* are always greater than *P*_0_.

We now consider the dynamics of the DSSA. If not stated otherwise, the initial molecular numbers of mRNA and protein are *M*(0) = 3 and *P*(0) = 100, respectively. We also make a comment about the scaling for mRNA and protein numbers that we use in the rest of this paper. In the Hirata et al. paper, it is clear that the data has been scaled but it is not clear what the scaling is. To be able to compare our results with those of Hirata et al. we perform one simulation with the values (*P*_0_,*h*,*τ*) = (100,4.1,19.7). We then use a scaling such that for this simulation the maximum amplitude of the mRNA is 4 and the protein is 7. This is consistent with the Hirata et al. data in their Figure 1. We then use this fixed scaling for all other DSSA simulations in this paper. The scaling factors for mRNA and protein are 0.3 and 0.03, respectively.

**Figure 4:**
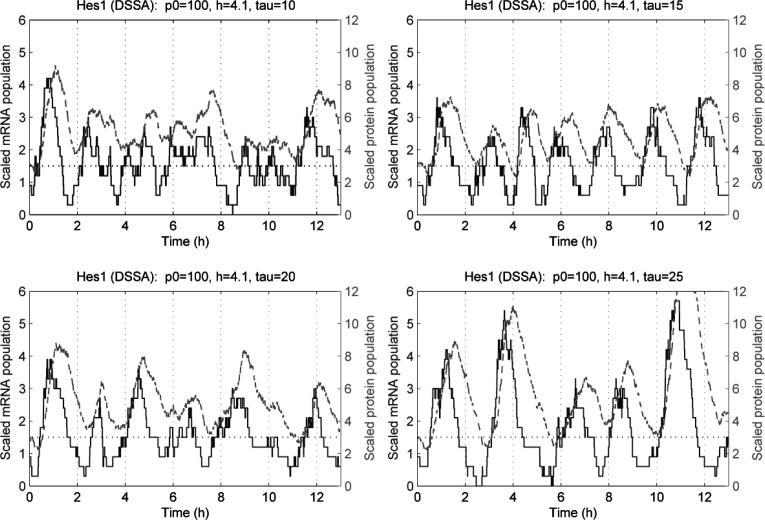
DSSA Simulations: Exploring the Effects of Delay The horizontal dotted line marks *P*_0_, mRNA is represented by the solid line, and protein by the dashed line.

In Figure 4 we keep *P*_0_ and *h* fixed and vary *τ* with *P*_0_ = 100, *h* = 4.1, *τ =* (10,15,20,25). We note that the oscillations are sustained and regular for *τ =* 15, 20, and 25, while there is some oscillatory behaviour even with *τ =* 5, but the dynamics are very irregular. Moreover, for all values of *τ,* protein numbers go mostly below *P*_0_, albeit for only small periods of time. However, for small delay (*τ =* 5) this happens far less often. Coincidentally or not, in this case the oscillations are very irregular. We also note that for larger values of *τ* (*τ =* 25), the amplitudes of protein concentration are generally larger than the values presented by Hirata et al.

**Figure 5:**
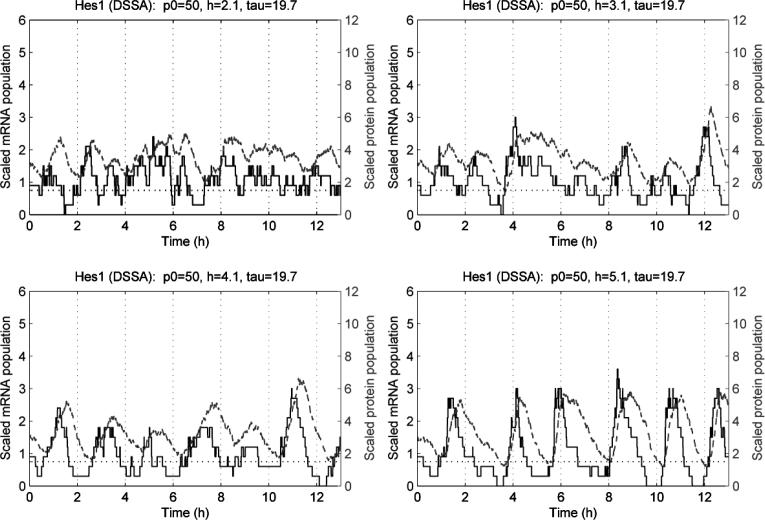
DSSA Simulations: Exploring the Effects of *h* The horizontal dotted line marks *P*_0_, mRNA is represented by the solid line, and protein by the dashed line.

In Figure 5 we perform a similar set of experiments but now we keep the delay fixed and vary the values of *h* with *P*_0_ = 50, *h* = (2.1,3.1,4.1,5.1), *t* = 19.7. Again, sustained and more or less regular oscillations are noticed for *h* = 4.1 and 3.1, but more irregular behaviour with *h* = 2.1 and 5.1.

**Figure 6:**
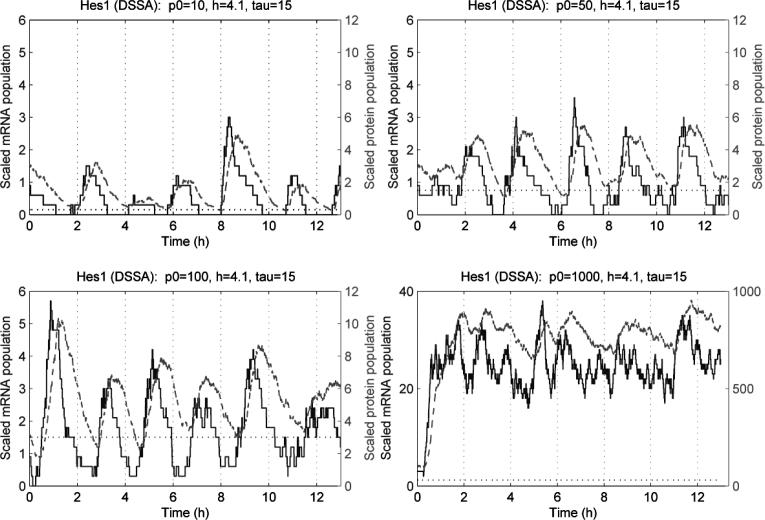
DSSA Simulations: Exploring the Effects of *P*_0_ The horizontal dotted line marks *P*_0_, mRNA is represented by the solid line, and protein by the dashed line.

Simulations in Figures 4 and 5 are performed with (*P*_0_ = 100) and (*P*_0_ = 50), respectively. Since the values of *P*_0_ might well be a significant factor in determining the dynamics of the system, we simulate also with varying *P*_0_ (10,50,100,1000) choosing parameters (*h*,*τ*) = (4.1,15) (Figure 6). We observe sustained, regular oscillations for *P*_0_ = (10,50,100). However, if *P*_0_ is very large (*P*_0_ = 1,000), the oscillations are very irregular and the numbers of protein are much larger than in the other cases. On the other hand, if *P*_0_ is low (*P*_0_ = 10), the amplitudes of mRNA and protein are not as large as in the other cases.

**Figure 7:**
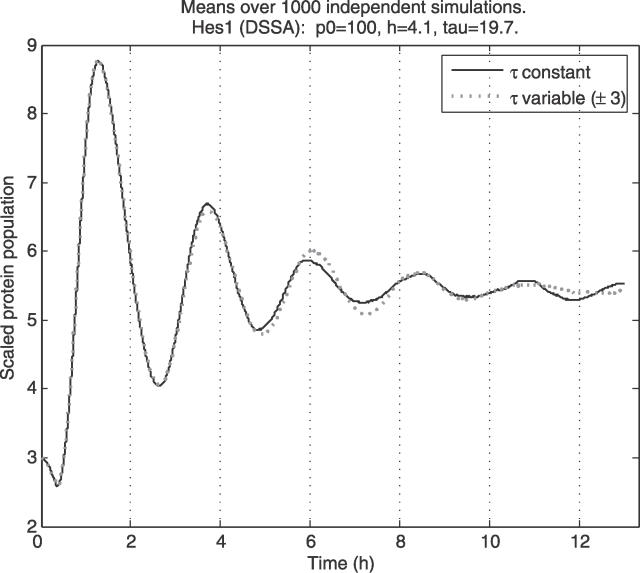
DSSA Simulation: Calculating the Arithmetic Mean over 1,000 Simulations for Both Constant and Variable Delay

The data shown by Hirata et al. represents the average of the samples from a number of cells. We computed the time-dependent arithmetic mean over 1,000 independent simulations using the DSSA with *P*_0_ = 100, *h =* 4.1, and *τ =* 19.7 (Figure 7). By comparing the result with the solution of the corresponding DDE (that is derived in Text S2), the following two aspects become evident. First, in spite of the differences between individual simulations due to the inherent stochasticity, the arithmetic mean over 1,000 independent simulations with either constant delay or variable delay is very close to the corresponding solutions of the DDE. Second, there appears to be no qualitative difference between the arithmetic mean of DSSA with constant delay compared with a uniformly distributed delay in an interval of width 6 centred around the bifurcation point of 19.7. The reason why the oscillation dies away is not that the system does not oscillate any more. Rather, the oscillations show a progressive, increasingly randomly distributed phase shift cancelling each other.

In addition, by performing numerous simulations for values (*P*_0_,*h*,*τ*) = (100,4.1,19.7) with initial conditions (*M*(0), *P*(0)) = (1,1), we searched for the occurrence of overshoot. The resulting trajectories did not show significant overshoot in the system, indicating that overshoot is not an issue for model 1 in the DSSA regime.

### Experimental Comparison

In this subsection we compare our simulations with specific experiments performed by Hirata et al. One of the most important aspects of the Hirata data is the regularity of the oscillatory period, which is 2 h. We therefore performed a spectrum analysis (more than 300 independent simulations) that takes a signal in the time domain and transforms it into its component frequency representation (frequency analysis has been done using a software package provided by Barrio et al., see Acknowledgements).

**Figure 8:**
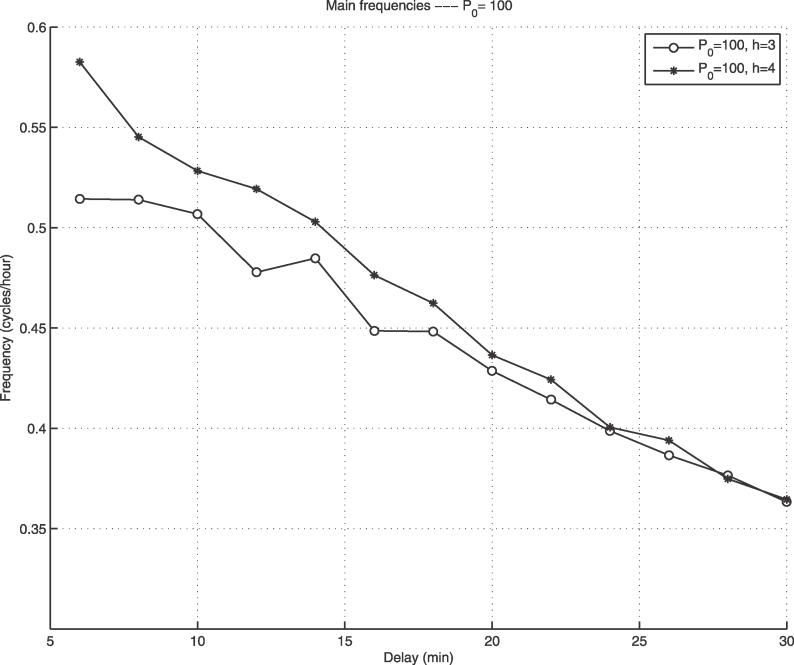
Mean Oscillation Frequencies for Different Delay Values (*P*_0_, *h*, *μ_m_, μ_p_* Are Fixed)

**Figure 9:**
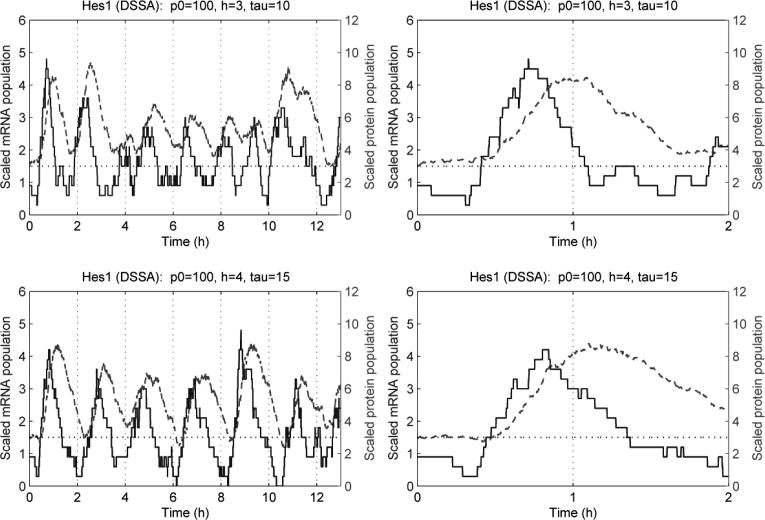
DSSA Simulations for *P*_0_ = 100, *h* = 3, *τ* = 10 (Top) and *P*_0_ = 100, *h* = 4, *τ* = 15 (Bottom) over 12 h and 2 h Left, more than 12 h. Right, more than 2 h. The dotted horizontal line corresponds to *P*_0_. Solid lines represent mRNA, dashed lines protein populations.

The oscillation frequencies can be determined for different values of the parameters: *P*_0_, *h*, *τ*, and the degradation rates. For each set of values the mean frequency of all the simulations is calculated. Figure 8 illustrates results for two cases where we seem to get regular dynamics in the oscillations, namely *P*_0_ = 100, *h* = (3,4). In both of these cases the frequency is plotted against the delay in the range [6,30]. It shows that the frequency decreases more or less linearly with the delay. This has a very important meaning for the oscillatory nature of the Hes1 regulatory system. It also confirms that the single snapshots of trajectories that we present in this paper do indeed capture the significant dynamics of the Hes1 model. We can conclude that for an oscillatory period of 2 h (frequency = 0.5), then if *h* = 3 an appropriate value for *τ* is about 10 while if *h =* 4 an appropriate value for *τ* is about 15. Of course, these relationships between *h* and *τ* are not completely precise, as simulations with *h =* 4 and *τ =* 19.7 (Figure 5) still show very regular behaviour with a period close to 2 h. Nevertheless, taking (*h*,*τ*) = (3,10) and (4,15) as being very appropriate values, we then performed a single simulation over 12 h (Figure 9, left plots). The first 2 h of each plot are shown separately (Figure 9, right plots). The simulations obtained compare very favourably with the Hirata et al. data in terms of the regularity of the period (2 h), the amplitude of the profiles, and the time lag of approximately 15–18 min (seen best from the simulations over 2 h).

**Figure 10:**
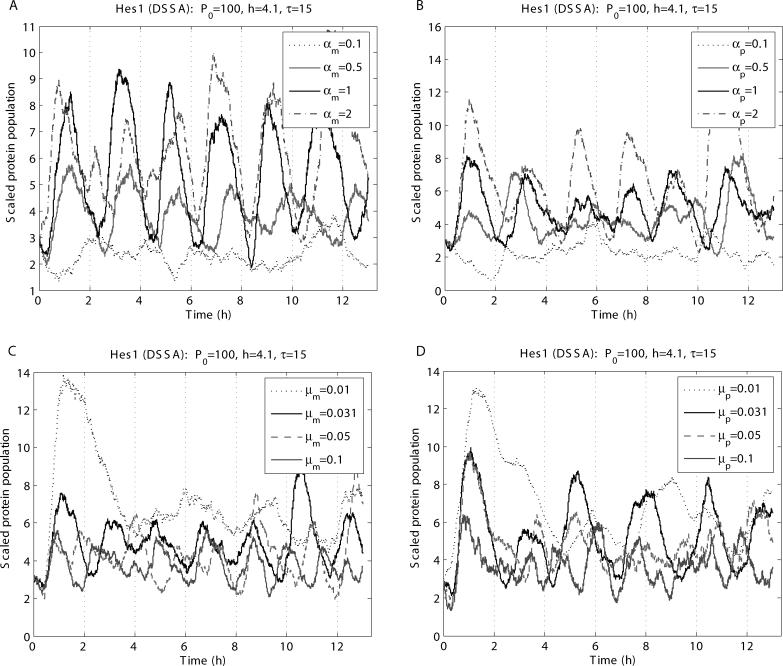
Sensitivity Analysis: DSSA Simulations with Perturbed Rate Constants (A) *α_m_* **.** (B) *α_p_* **.** (C) *μ_m_* **.** (D) *μ_p_*.

By perturbing the reaction rate constants, we can attempt to get an idea of the system's sensitivity (we note that Hirata et al. observed alterations in the oscillatory period as the temperature was lowered). This approach does not replace a thorough analysis. However, it still leads to insights about the different dynamics and the sensitivity of the model. Figure 10 shows DSSA trajectories for simulations with parameters *P*_0_ = 100, *h* = 4.1, *τ* = 15, and one of the four reaction rate constants *α_m_*, *α_p_*, *μ_m_*, and *μ_p_* varied in each figure while the others are kept fixed with values as shown in Table 1 *(α_m_ = 0*.1,0.5,1,2, *α_p_ =* 0.1,0.5,1,2, *μ_m_ =* 0.01,0.029,0.05,0.1, and *μ_p_ =* 0.01,0.031,0.05,0.1). Perturbing *α_m_* or *α_p_* in these ranges leads to similar dynamical behaviour: oscillations become more regular, with larger amplitudes and shorter periods, the larger the production rate constant. Oscillations are barely visible for *α_m_ = α_p_ =* 0.1. For *α_m_ = α_p_ =* 0.5, we obtain regular oscillations (although the trajectory for *α_p_ =* 0.5 in Figure 9 does not look very regular). For *α_m_* = *α_p_ =* 2, oscillations are very regular with high peaks and short period. By varying the value of *μ_m_*, we observe very irregular dynamics for low degradation rates (0.01) and regular oscillations with larger rates (0.029, 0.05). However, for even larger degradation rates (0.1), oscillations become irregular again. The average scaled population level decreases with *μ_m_* increasing. For *μ_p_,* we observe the opposite behaviour, namely that oscillations occur for all four values of *μ_p_,* but that the period increases and peaks decrease in size as *μ_p_* becomes smaller. Small perturbations around (*α_m_ =* 1, *α_p_ =* 1, *μ_m_ =* 0.029, *μ_p_ =* 0.02) do not seem to have any visible effect on the oscillatory dynamics. Because data is not available on how a reduction in temperature from 37 °C to 30 °C affects the period of oscillation, we are unable to compare our simulations with experimental results, but our simulation results are not unreasonable.

Finally, we compare the results of some actual experiments by Hirata et al. with the corresponding modified DSSA simulations. The experiment in which Hes1 protein degradation is blocked by application of proteasome inhibitor MG132 is mimicked by setting the fourth stoichiometric vector to *v*_4_ = (0,0)*^T^,* thus making the protein degradation reaction ineffective. This happens at a predefined time *t_exp_* after oscillation is initiated. Figure 10 illustrates the effect on the model for parameters *h =* 4.1, *τ =* 15, *P*_0_ = 100, and *t_exp_ =* 60 min when protein degradation is blocked. Evidently, the dynamics of the modified model matches with those from the experiment quite well (Figure 3A in [3]). For the experiment in which translation is inhibited by cycloheximide treatment, we neutralize translation by setting the third stoichiometric vector at *v*3 = (0,0)*^T^*. As with the first experiment, the modification is set off at a predefined time after simulation starts. Figure 10 shows a typical trajectory resulting from a DSSA run with parameters *h =* 4.1, *τ =* 15, *P*_0_ = 100, and *t_exp_ =* 30 min when translation is blocked. The model performs in much the same way as described in the experiment (Figure 3C in [3]).

## Discussion

When we compare the dynamics of DSSA with the continuous delay case, we can make a number of important conclusions. Perhaps the most significant is that there are sustained oscillations for values of *h* < 4.1 and *τ* < 19.7, unlike the continuous case. Indeed, Figure 5 shows sustained regular oscillations with values of *h* about 3. This is an important point. In the continuous setting, there is a large set of Hill functions that represent a wide variety of bindings of molecules to operator regions. Modellers infer information about the nature of the cooperativity at the binding sites from the dynamics of simulations of models with different types of Hill functions. What our simulations show is that these values may be overestimated from continuous models and that discrete delay models may well give more realistic values for cooperativity. Furthermore, it is clear that with our model if the value of *h* is too small, certainly *h* < 2, then the oscillations are very noisy and very irregular. Indeed, Figure 8 strongly suggests that for an oscillatory period of two hours to occur, *h* cannot be much less than 3.

The same remarks apply for estimates of the values of *τ* that lead to sustained regular oscillations. Thus from Figure 9 we observe reasonably well-defined sustained regular oscillations for values of *τ =* 15 with *h =* 4, and *τ =* 10 with *h =* 3. Values for *τ* lower than *τ =* 10 result in noisy and irregular delay. On the other hand, values of *τ* bigger than 20 suggest an *h* larger than 5, and this seems to be too high a value for the Hill parameter in terms of the number of operator binding sites. For values of (*P*_0_,*h*,*τ*) = (100,3,10) and (100,4,15), the simulations in Figure 9 compare very favourably with the data given by Hirata et al. (their Figure 1). The oscillations are regular with a period of 2 h, the amplitudes have more or less the same values in the simulations, and the experiments and the time lag of about 15–18 min, seen in the rightmost plots of Figure 9, is very close to that observed by Hirata et al.

Another feature of the dynamics of DSSA that we would like to emphasise is the role of *P*_0_. For continuous models, the role of *P*_0_ appears not to be too significant as long as it is not too large. But from Figure 6 we see that *P*_0_ plays an important role. Apparently, when the numbers of *P* are below *P*_0_, there is expression. This expression only occurs for very small time windows but seems to be crucial in driving the oscillations. This behaviour does not occur for the continuous deterministic models. If the value of *P*_0_ is increased too much to *P*_0_ = 1,000, say, then there are no oscillations and *P* never goes below *P*_0_. On the other hand, if *P*_0_ is too low (*P*_0_ = 10), then the amplitudes of the mRNA and protein appear to be too small. This provides a prediction that should be able to be tested experimentally.

Furthermore, simulations in Figures 4–6 and 9 suggest that the peak-to-trough ratios of mRNA and protein are in closer agreement to the Hirata et al. data than those obtained from the deterministic model. The Hirata data suggests ratios of between 3 and 4 for mRNA and between 3 and 8 for protein, although we note that these only approximate values as there are significant error bars for the protein concentrations. On the other hand, a rough estimate from the discrete simulations gives a peak-to-trough ratio for protein of between 3 and 4 with a larger ratio for mRNA due to the fact that the numbers of mRNA can become quite small in some cases.

We have also shown from the mathematical analysis in the supporting information (Text S2) that the mean behaviour of the DSSA is well-described by the corresponding DDE when there are large numbers of molecules. This naturally generalizes the SSA/DDE case and provides a comprehensive framework for studying noise in biology.

Furthermore, our simulations suggest that overshoot is not an issue for model 1 in a discrete delay setting. One of the reasons that Bernard et al. [11] introduced model 2 in the continuous delay setting was because overshoot is not so pronounced when there are more nonlinearities in the model. However, in the discrete setting, these differences appear not to be significant, and so choosing a model based on overshoot issues appears not to be important.

**Figure 11:**
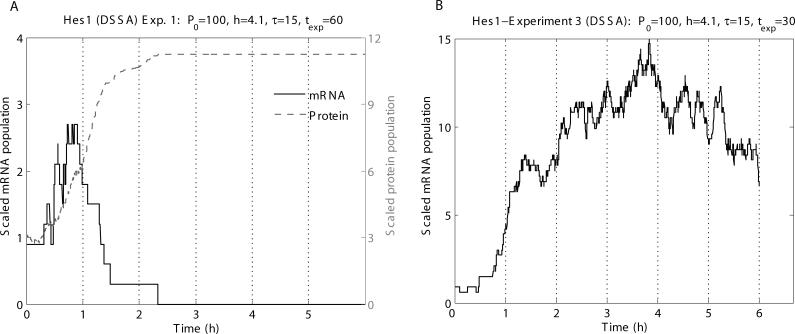
DSSA Simulation (A) Mimicking blocking of Hes1 protein degradation by a proteasome inhibitor. (B) Mimicking inhibition of translation by cycloheximide treatment.

The sensitivity analysis in Figure 10 shows that the DSSA is more sensitive to the degradation parameters than the production parameters in terms of their effect on the period of oscillation. However, there is more sensitivity of the discrete model to the production parameters than for the continuous model 1. Moreover, the DSSA simulations in Figure 11 mimic very well the experiments when either protein degradation or translation is blocked, both in terms of the time of the degradation of mRNA numbers and in actual values of mRNA and protein.

Putting all this information together we see that we get very good comparisons between simulation and experiment if the value of *P*_0_ is on the order of 50 to 100, if the value of *h* is somewhere between just less than 3 to just bigger than 4, and if the delay is somewhere between 10–20 min We can be more precise if more accurate values of the transcription and translation delays are available. If this sum of delays is about 15, then this suggests a Hill factor of about 4, while if the delay is about 10, then a Hill factor of 3 is more appropriate.

### Conclusions

In this paper we have compared continuous delay models and discrete, stochastic delay models to explain oscillations in numbers of *hes1* mRNA and Hes1 protein in mouse. Given that the numbers of mRNA and proteins produced are relatively small, the discrete delay approach may well be more appropriate than the continuous approach. Furthermore, the discrete delay approach seems to give greater insight into the underlying cellular dynamics in terms of the system parameters.

By careful comparisons of our simulations with the Hirata et al. data, we have been able to suggest quite specific ranges for *P*_0_, the Hill parameter *h*, and the delay *τ*. We have also shown by both mathematical analysis and simulations that the mean behaviour of DSSA is described by a DDE. This naturally generalises the nondelay case and provides a comprehensive and consistent mathematical framework for understanding the role of noise in biology.

## Supporting Information

Text S1 DSSAs (89 KB PDF)

Text S2 DDEs and the Master Equation (45 KB PDF)

### Accession Numbers

The Entrez Gene (http://www.ncbi.nlm.nih.gov/entrez/query.fcgi?DB=gene) gene ID for the hes1 gene in Mus musculus (Mouse) is 15205. The Swiss Prot (http://www.ebi.ac.uk/swissprot/) corresponding accession number for the Hes1 protein is P35428.

## Abbreviations

CME: chemical master equation
DDE: delay differential equations
DSSA: delay stochastic simulation algorithm
SSA: stochastic simulation algorithm
