# Supplementary material for: Oscillatory Regulation of Hes1: Discrete Stochastic Delay Modelling and Simulation
Source: PLoS Comput Biol. 2006 Sep 8;2(9):e117. doi: 10.1371/journal.pcbi.0020117 (PMC1560403; doi:10.1371/journal.pcbi.0020117)
Supplement: Supplementary file 1 [file pcbi.0020117.se001.pdf]

Supplementary Text S1:

Delay Stochastic Simulation Algorithms (DSSAs)

This contribution presents a discussion on different algorithms performing a delayed stochastic simulation. As our simulations show, for the Hes1 model (cf. model 1 in the paper) these implementations are more or less equivalent, and indicates the robustness of the particular model to these nuances in implementation.

The SSA is predicated on the assumption that there is only one reaction occurring per step. Other techniques such as the τ-leap methods [5, 6] allow a number of reactions per step. However, in this case we lose information about possibly important orderings of the reactions. By including delayed reactions into the SSA the evolution of the system becomes a non-Markovian process and simultaneousness of events becomes an issue the DSSA has to deal with as non-delayed, instantaneous reactions occur while delayed reaction wait for being updated.

The DSSA versions discussed in this supplement differ in the way they deal with the following three simulation aspects:

1. **Waiting time.** In the SSA the time between two reactions is regarded as the waiting time until the next reaction occurs, while reactions happen instantaneously [4]. In the DSSA the waiting time for delayed reactions is either determined in the same way as the time step for non-delayed reactions (according to the SSA) or it is ignored (see DSSA 1). In the latter case, we might argue that the waiting time is already incorporated in the delay time. However, separating waiting time and delay seems to be more appropriate as this is a more natural representation of chemical kinetics.

2. **Time steps in the presence of delayed reaction updates.** Updates of delayed reactions can change the system significantly. We can take this into account with the following strategy: the DSSA proceeds in the same way as the SSA as long as there are no delayed reactions scheduled in the next regular time step but moves forward to the scheduled delayed reaction otherwise. However, in doing so we ignore the selected reaction that should be updated beyond the current update point. This can be considered as changing the stochastic path as it is equivalent to choosing a random variable and then rejecting it. There is a number of papers in the literature that stress the importance of maintaining the Brownian path when solving stochastic problems [2, 3]. If the next step is just determined according to the updated state we also ignore the elapsed time since the last reaction was triggered. It is unclear whether this affects the distribution of waiting times until the next reaction happens.

Another approach simply updates the scheduled delayed reactions with the next non-delayed reaction that is selected (see DSSA 2). This method ignores any changes of the system's state due to the delayed reactions within this time step. Also, by doing so we lose the property of only one reaction per time step. We note that this approach is fundamentally different to the τ-leap method for the SSA because it allows only one non-delayed update per time-step.

Both approaches imply that the exactness of the SSA cannot be transferred to the corresponding DSSA implementations.

3. **Updating delayed consuming reactions.** The system's state at the moment of selection of the delayed reaction can be very different to the state at the moment when the delayed reaction is updated, as delays due to transcription and translation can be large. When in the period between selection and update of a delayed consuming reaction other reactions occur that consume the same reactants, the molecular number of those reactants can become negative by updating the delayed reaction. Therefore, reactants and products of delayed consuming reactions must be updated separately — namely when the delayed reaction is selected and when it is completed, respectively (see DSSA 4). In case the delayed reactions are all non-consuming this aspect can be ignored.

The first DSSA version is proposed in [1]. Figure 1(a) illustrates the scheme and its working in terms of the three issues mentioned above. This algorithm ignores the waiting time (1) and works only for non-consuming reactions (3) since there is only one update when a delayed reaction is due. In terms of the time steps (2), it preferentially updates delayed reactions and thus, at some points selects but ignores a reaction if the time step would pass the update points of a delayed reaction. This implies that there is ever only one reaction per step, whether this is delayed or non-delayed, and is similar to SSA in this respect.

The second DSSA (Figure 1(b)) includes waiting times also for delayed reactions (1). It was specifically designed to work for the Hes1 model (where the delayed reaction is a non-consuming reaction) and, thus does not consider consuming reactions (3). Moreover, the delayed reactions that are scheduled at time points passed by a simulation step are updated all together with the latest reaction (2).

The third DSSA (Figure 1(c)) comprises the attributes of the first and the second algorithm as it considers waiting times (1) and updates only one reaction per step by updating delayed reactions when they are due and ignoring the reaction that is selected for the time step (3). However, it still does not run with delayed consuming reactions. This deficiency is remedied with the fourth DSSA (Figure 1(d)).

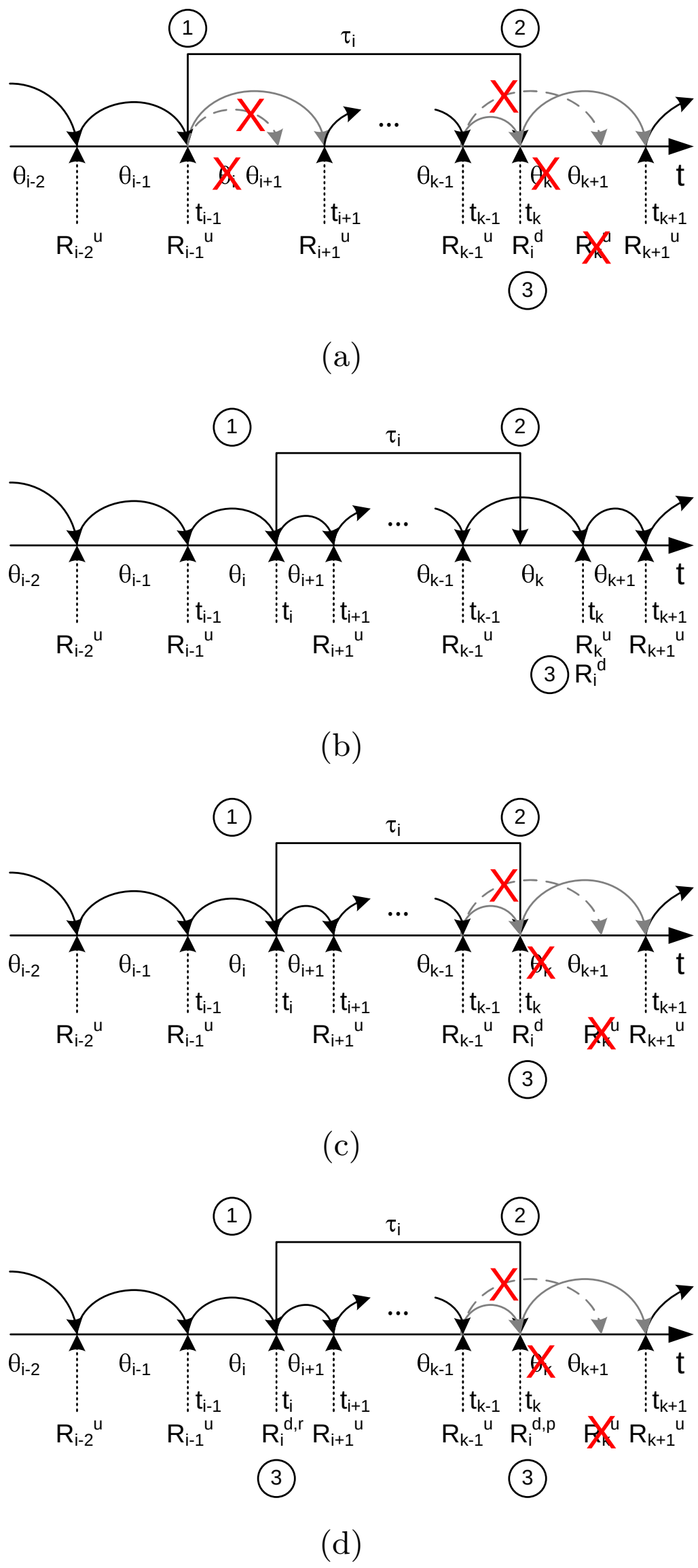

(a) (b) (c) (d)

Figure 1: Four different DSSA implementations. The numbers 1–3 refer to the three aspects and appear where these aspects become visible in the schemes. θ_i refers to the waiting times until the next reaction R_i is scheduled and τ_i is the delay of reaction R_i. The dotted arrows point to the time line indicating when a reaction is updated. The reaction is specified below the arrow. R_i^u denotes the non-delayed reaction triggered and updated at time t_i and R_i^d the delayed reaction that is triggered at time t_i. In case reactants and products of a delayed reaction are separately updated this is marked by $R_i^{d,r}$ and $R_i^{d,p}$, respectively. If time steps and reactions are drawn but then ignored they are crossed out. Those steps are marked as grey dashed lines and the steps replacing an ignored step are marked as grey solid lines.

As emphasised previously, for our particular model there is very little difference in the DSSA implementations but these issues could be important in other settings. In the paper all simulations are run with the third DSSA. In the Supplementary Text S2 we derive a chemical master equation ansatz for the third DSSA.

References

[1] Bratsun D, Volfson D, Tsimring LS, Hasty J (2005) Delay-induced stochastic oscillations in gene regulation. Proc Natl Acad Sci USA 102:14593-14598.

[2] Burrage PM, Burrage K (2002) A variable stepsize implementation for stochastic differential equations. SIAM J Sci Comput 24(3):848–864.

[3] Gaines JG, Lyons TJ (1997) Variable stepsize control in the numerical solution of stochastic differential equations. SIAM J Appl Math 5:1455–14884.

[4] Gillespie DT (1977) Exact stochastic simulation of coupled chemical reactions. J Phys Chem 81:2340-2361.

[5] Gillespie DT (2001) Approximate accelerated stochastic simulation of chemically reacting systems. J Chem Phys 115:1716-1733.

[6] Tian T, Burrage K (2004) Binomial leap methods for simulating stochastic chemical kinetics. J Chem Phys 121:10356-10364.
