# Supplementary material for: Oscillatory Regulation of Hes1: Discrete Stochastic Delay Modelling and Simulation
Source: PLoS Comput Biol. 2006 Sep 8;2(9):e117. doi: 10.1371/journal.pcbi.0020117 (PMC1560403; doi:10.1371/journal.pcbi.0020117)
Supplement: Supplementary file 2 [file pcbi.0020117.se002.pdf]

# Supplementary Text S2:

## Delay Differential Equations and the Master Equation

The SSA describes the evolution of a discrete nonlinear Markov process. This stochastic process has a probability density function that is the solution of a differential equation (the Chemical Master Equation). This master equation can be used to write down an ODE that describes the deterministic behaviour of the mean associated with the SSA or a stochastic differential equation (SDE) that represents the intrinsic noise in continuous form. In a similar manner the DSSA described in section 3 will have corresponding representations as delay differential equations (DDEs) or stochastic delay differential equations (SDDEs).

The discussion in this section is aimed at providing the theoretical background for constructing DDEs that faithfully represent the mean behaviour of intrinsic noise (in a continuous form) arising from delayed reactions. Here it is assumed that a well-stirred chemical reaction system contains $N$ molecular species $\{S_1, \ldots, S_N\}$ with number $X_i(t)$ of the species $S_i$ at time $t$. Among the $M$ reaction channels, the first $M_1$ reactions $\{R_1, \ldots, R_{M_1}\}$ are assumed to have time delay $\{T_1, \ldots, T_{M_1}\}$, respectively, and the last $M - M_1$ reactions have no time delay. From the description of the DSSA in section 3, the state vector $X(t)$ is a non-negative $N$-dimensional jump stochastic process but is not a Markov process any more due to the time delay. Here we are interested in the conditional probability function based on the initial state $\mathbf{X}(t_0)$ and the states involved with the time delay $\mathbf{X}(t) = \Phi(t)$ $(t \leq t_0)$, given by

$$P(\mathbf{x}, t) \equiv \text{Prob}\{\mathbf{X}(t) = \mathbf{x} | \mathbf{X}(t_0) = \mathbf{x}_0, \text{ and } \mathbf{X}(t) = \Phi(t), \ t \leq t_0\}. \tag{1}$$

For Discrete Chemical Kinetic models with time delay, the master equation should be based on the current system state at time $t$ for reactions without time delay and the system state at $t - T_j$ for the reaction channel $R_j$ with time delay $T_j$. In order to derive the time evolution equation of the probability function (1), we take a time increment $dt$ that is so small that the probability for two or more reactions to occur in $dt$ is negligible compared to the probability for at most one reaction. It is assumed that the reaction time of a reaction without time delay is $dt$, while for a reaction with time delay $T_j$ the reaction time is $dt + T_j$. Then the probability of the system being in state $\mathbf{x}$ at $t + dt$ is given by

$$
\begin{aligned}
P(\mathbf{x}, t+dt) &= P(\mathbf{x}, t) - \sum_{j=1}^{M_1} \sum_{\mathbf{x}_i \in I(\mathbf{x})} a_j(\mathbf{x}_i) P(\mathbf{x}, t; \mathbf{x}_i, t-T_j) dt \\
&+ \sum_{j=1}^{M_1} \sum_{\mathbf{x}_i \in I(\mathbf{x})} a_j(\mathbf{x}_i) P(\mathbf{x}-\nu_j, t; \mathbf{x}_i, t-T_j) dt \\
&- \sum_{j=M_1+1}^{M} a_j(\mathbf{x}) P(\mathbf{x}, t) dt + \sum_{j=M_1+1}^{M} a_j(\mathbf{x}-\nu_j) P(\mathbf{x}-\nu_j, t) dt,
\end{aligned} \tag{2}
$$

where $P(\mathbf{x}, t; \mathbf{x}_i, t-T_j)$ is the probability that the system is both in the state $\mathbf{x}$ at $t$ and in the state $\mathbf{x}_i$ at $t-T_j$, and $I(\mathbf{x})$ is the set of all possible system states. The second term on the right hand side is the probability that no reaction will fire in $[t-T_j, t+dt)$ for the reaction $R_j$ with time delay $T_j$ and the system is in the state $\mathbf{x}$ at $t+dt$. Here we should consider all the possible states $\mathbf{x}_i$ at $t-T_j$ because the system evolves in the time period $[t-T_j, t)$ based on the reactions of other reaction channels. For the case of one reaction, the third term also considers all the possible system states at $t-T_j$ for the reaction with time delay $T_j$ but the system should be in the state $\mathbf{x}-\nu_j$ at $t$. The last two terms are the probabilities for reactions without time delay, in which only the system states at $t$ should be included in the master equation.

When $dt \to 0$, this leads to the delay chemical master equation

$$
\begin{aligned}
\frac{\partial}{\partial t} P(\mathbf{x}, t) &= -\sum_{j=1}^{M_1} \sum_{\mathbf{x}_i \in I(\mathbf{x})} a_j(\mathbf{x}_i) P(\mathbf{x}, t; \mathbf{x}_i, t-T_j) \\
&+ \sum_{j=1}^{M_1} \sum_{\mathbf{x}_i \in I(\mathbf{x})} a_j(\mathbf{x}_i) P(\mathbf{x}-\nu_j, t; \mathbf{x}_i, t-T_j) \\
&- \sum_{j=M_1+1}^{M} a_j(\mathbf{x}) P(\mathbf{x}, t) + \sum_{j=M_1+1}^{M} a_j(\mathbf{x}-\nu_j) P(\mathbf{x}-\nu_j, t).
\end{aligned} \tag{3}
$$

The value of $a_j(\mathbf{x}_i) P(\mathbf{x}-\nu_j, t; \mathbf{x}_i, t-T_j)$ is the probability that one reaction $R_j$ (with time delay $T_j$) fires at $t-T_j$ and at the same time the system states are $\mathbf{x}-\nu_j$ at time $t$ and $\mathbf{x}_i$ at time $t-T_j$, respectively. Here the probability $P(\mathbf{x}-\nu_j, t; \mathbf{x}_i, t-T_j)$ measures the strength of coupling of the system states at $t$ and $t-T_j$. If the number of reactions in the time period $[t-T_j, t]$ is relatively small, this probability is critical to the system behaviour and the DSSA must be employed. However, if the time delays are large and there are a relatively large number of reactions in the time interval $[t, t-T_j)$, it is reasonable to assume that the coupling of the system

states at $t$ and $t - T_j$ is weak and the probability can be approximated by

$$P(\mathbf{x} - \nu_j, t; \mathbf{x}_i, t - T_j) \approx P(\mathbf{x} - \nu_j, t) \times P(\mathbf{x}_i, t - T_j). \qquad (4)$$

Here we should re-emphasize that the above assumption is based on the fact that there are a relatively large number of reactions firing in the time period $[t - T_j, t]$, which is the case being considered here. In this case all the different DSSA variants discussed in Supplementary Text S1 can be considered equivalent.

Based on assumption (4), we can obtain the mean of the propensity functions for reactions with time delay, given by

$$\overline{a_j(\mathbf{x}(t - T_j))} = \sum_{\mathbf{x}_i \in I(\mathbf{x})} a_j(\mathbf{x}_i) P(\mathbf{x}_i, t - T_j). \qquad (5)$$

Then the delay chemical master equation can be simplified as

$$\begin{aligned} \frac{\partial}{\partial t} P(\mathbf{x}, t) &= -\sum_{j=1}^{M_1} \overline{a_j(\mathbf{x}(t - T_j))} P(\mathbf{x}, t) + \sum_{j=1}^{M_1} \overline{a_j(\mathbf{x}(t - T_j))} P(\mathbf{x} - \nu_j, t) \\ &- \sum_{j=M_1+1}^{M} a_j(\mathbf{x}) P(\mathbf{x}, t), + \sum_{j=M_1+1}^{M} a_j(\mathbf{x} - \nu_j) P(\mathbf{x} - \nu_j, t). \qquad (6) \end{aligned}$$

In the case that there is no delay equation (6) reduces to the well known Chemical Master Equation associated with the SSA, namely

$$\frac{\partial}{\partial t} P(\mathbf{x}, t) = \sum_{j=1}^{M} a_j(\mathbf{x} - \nu_j) P(\mathbf{x} - \nu_j, t) - \sum_{j=1}^{M} a_j(\mathbf{x}) P(\mathbf{x}, t). \qquad (7)$$

If we multiply the delay chemical master equation (6) through by all of the states at $t$, sum over all these system states, and then re-index the summation on the right-hand side, we can obtain the equations for the mean of $\mathbf{X}(t)$

$$\frac{d\overline{\mathbf{X}(t)}}{dt} = \sum_{j=1}^{M_1} v_j \overline{a_j(\mathbf{X}(t - T_j))} + \sum_{j=M_1+1}^{M} v_j \overline{a_j(\mathbf{X}(t))}. \qquad (8)$$

When all molecular numbers are very large and fluctuations are not important, we can get the delay reaction rate equation, given by

$$\frac{d\mathbf{X}(t)}{dt} = \sum_{j=1}^{M_1} v_j a_j(\mathbf{X}(t - T_j)) + \sum_{j=M_1+1}^{M} v_j a_j(\mathbf{X}(t)). \qquad (9)$$

Note, if there are no delays, (9) is an ODE describing the standard chemical kinetics rate equations.

We note that we can use quasi steady-state assumptions [1, 2, 3] that may encapsulate detailed chemical kinetics within Hill functions and use these Hill functions in place of the simpler propensity functions. Then, this will lead directly to the model that Monk considered.

## References


[1] Haseltine EL, Rawlings JB (2002) Approximate simulation of coupled fast and slow reactions for stochastic chemical kinetics. J Chem Phys 117:69596969.

[2] Rao C, Arkin A (2003) Stochastic chemical kinetics and the quasi-steady-state assumption: Application to the Gillespie algorithm. J Chem Phys 118:4999-5010.

[3] Tian T, Burrage K (2005) Stochastic Models for Regulatory Networks of the Genetic Toggle Switch. Proc Natl Acad Sci USA. Submitted.
